# A Cooking Intervention to Increase Vegetable Consumption by Parents With Children Enrolled in an Early Head Start Home Visiting Program: A Pilot Study in Portland, Oregon, 2013–2014

**DOI:** 10.5888/pcd13.160259

**Published:** 2016-12-22

**Authors:** Betty T. Izumi, Cara L. Eckhardt, Dara P. Wilson, Jennifer Cahill

**Affiliations:** 1 Oregon Health & Science University–Portland State University School of Public Health, Portland, Oregon; 2 Mt. Hood Community College Head Start and Early Head Start, Portland, Oregon.

## Abstract

**Introduction:**

Cooking interventions may improve diet quality. Most cooking interventions are delivered in group settings. Home visiting programs may be an appropriate mechanism for delivering such interventions to low-income families with young children. We conducted a pilot study to test the feasibility of using a cooking intervention delivered by home visitors to improve attitudes and behaviors related to vegetable consumption by low-income parents with children enrolled in a home visiting program.

**Methods:**

We invited 121 parents with children enrolled in an Early Head Start Home Visiting program in Portland, Oregon, to participate. During 2013–2014, each month for 8 months, home visitors (n = 14) implemented 1 cooking activity plus 1 complementary activity focused on 12 vegetables. We collected pre- and post-intervention data on participants’ cooking confidence and whether they tried and liked the selected vegetables. We also measured fidelity to protocol and home visitors’ perception of intervention usability.

**Results:**

Of 104 participants, 58 provided pre- and post-intervention data. We observed a significant increase in confidence in baking, roasting or grilling vegetables; cooking 6 of 10 vegetables; and trying 7 of 12 vegetables. Nearly all respondents participated in the monthly cooking activity (96%) and complementary activity (94%). Twelve of 14 home visitors reported that the intervention was acceptable, feasible, and easy to understand, and needed systems supports to implement.

**Conclusion:**

Cooking interventions may be a feasible approach to improving attitudes and behaviors related to vegetable consumption by low-income families with young children. Additional research is needed to assess the impact of such interventions on vegetable consumption.

## Introduction

The benefits of consuming a diet rich in vegetables are well documented (1–3), yet most Americans, particularly those from low-income households, do not meet current recommendations for vegetable consumption: 2 to 3 cups of vegetables per day for adults aged 19 years or older (4–7). Because food preferences are developed early in life (8,9), early interventions to promote vegetable consumption are critical. During the first few years of life, parents or other primary caregivers (hereinafter called parents) have the greatest influence on the development of their children’s eating behaviors. Interventions designed to improve vegetable consumption by young children, therefore, should target their parents.

Cooking interventions are increasing in popularity because of their potential to improve diet quality (10,11). To date, most cooking interventions, including those that target low-income families with young children (11–14), have been delivered in group settings. Such families, however, can be difficult to engage in group interventions because of barriers such as lack of reliable transportation and inconsistent work schedules (14,15). Home visiting, a long-standing and successful method of delivering parenting education and services to hard-to-reach families (eg, low-income, teenaged parents) with young children (16–20), may be an effective mechanism for engaging this population in cooking interventions. Home visiting reaches families in the comfort of their homes and can provide them with experiences that meet their individual needs (15,21). In fiscal year 2015, more than 145,000 parents and children nationwide received services through evidence-based home visiting models supported by the Federal Home Visiting Program (22). To date, no study has described the feasibility of integrating a cooking intervention into an existing home visiting program to improve attitudes and behaviors related to vegetable consumption by low-income families with young children.

The primary objective of this pilot study was to test the feasibility of using a cooking intervention delivered by home visitors through an Early Head Start Home Visiting program to improve confidence in cooking vegetables among low-income parents with children aged 0 to 3 years and to encourage families to try and learn to like eating vegetables. A secondary objective was to assess fidelity to program protocol and the home visitors’ perception of intervention usability.

## Methods

### Participants and study design

An Early Head Start Home Visiting program in Portland, Oregon, was selected for this pilot study because it was part of an existing community-based participatory research partnership with Mt. Hood Community College (MHCC) Head Start and Early Head Start, which operates programs based at Head Start and Early Head Start centers and in homes. Early Head Start Home Visiting is an evidence-based home visiting model that serves low-income pregnant women and families with children aged 0 to 3 years in households that have incomes below 185% of the federal poverty level. In this model, trained home visitors provide weekly 90-minute home visits focused on a range of child development activities. Study participants were parents whose children were enrolled in the participating Early Head Start Home Visiting program during the 2013–2014 academic year. Home visitors employed by the participating program also took part in this study.

All parents of children enrolled in the participating program (n = 121) received a letter in September 2013 inviting them to take part in this study. Of these parents, 104 (86%) elected to participate. We collected pre-intervention survey data from parents in September 2013. The intervention period was October 2013 through May 2014. Post-intervention survey data were collected in June 2014. Parents received a cooking tool (eg, baking sheet, spatula) on completion of each survey. Fourteen home visitors participated in this study. No incentives were offered to home visitors for their participation because it was considered to be part of routine program operations. All study procedures were approved by the Portland State University institutional review board.

### Intervention

A workgroup made up of program staff (ie, administrators and home visitors), a parent, and researchers adapted the Harvest for Healthy Kids curriculum (23,24), a farm-to-preschool curriculum for children aged 3 to 5 years in early care and education settings, for use in the Early Head Start Home Visiting program. The adapted curriculum included a cooking activity and complementary activities (ie, literacy, art, music and movement, or floor time [Box]) that could be implemented during a 90-minute home visit. The activities focused on the following 12 vegetables: carrots, pumpkin squash, butternut squash, delicata squash, spaghetti squash, sweet potato, potato, cabbage, turnip, rutabaga, parsnip, and beets. These foods were chosen because they were already being featured in the MHCC Head Start and Early Head Start center-based programs.

**Box. Examples of Activities Used in a Pilot Cooking Intervention Delivered by Home Visitors in an Early Head Start Home Visiting Program, Portland, Oregon, 2013–2014**

**Cooking**

Prepare orange glazed carrots, a vegetable dish made with boiled carrots. Allow parents to take the lead in preparing and cooking carrots and engage children in developmentally appropriate tasks (eg, toddlers can help to scrub carrots clean).

**Literacy**

Use potatoes to practice counting while reading *One Potato: A Counting Book of Potato Prints *by Diana Pomeroy.

**Art**

Create a butternut squash puzzle by cutting and pasting a large image of a butternut squash to a piece of cardboard. Cover with Mod Podge (Plaid Enterprises, Inc) and allow to dry. Cut image into developmentally appropriate number of pieces.

**Music and movement**

Sing and dance *to Flower, Leaves, Stems, and Roots*, an adaptation of the well-known children’s song, *Head, Shoulders, Knees, and Toes*, in which children touch their head for flowers, shoulders for leaves, legs for stems, and toes for roots.

**Floor time**

Place various types of vegetables into a brown paper bag. Ask family members to guess what types of vegetables are in the bag.

Each month during the 8-month intervention period, home visitors implemented the intervention activities with each family in their caseloads during 2 of their 4 home visits. During 1 home visit, home visitors implemented a cooking activity focused on a selected vegetable; during another visit, they implemented one of the study’s other 4 activities (Box) that focused on the same vegetable. The time spent on the activities varied according to the activity and interest of the parent.

Before the intervention began, home visitors participated in a 2-hour hands-on training session to learn how to implement the study activities. During the intervention period, home visitors also participated in 3 additional hands-on training sessions, which provided home visitors with an opportunity to develop their cooking skills and practice cooking with the selected vegetables. The training sessions were conducted as part of the annual health and nutrition training sessions required for the participating program to meet program performance standards. In addition to the hands-on training sessions, the home visitors were provided with the curriculum and supplies (including ingredients for cooking activities and books to read aloud) needed to implement the study activities.

### Measurement

*Outcome evaluation.* Demographic data were collected by the Early Head Start Home Visiting program as part of its enrollment process for new and returning children. Demographic variables were the following: child’s age and parent’s age, sex, ethnicity, race, employment status, educational attainment, and participation in supplemental food assistance programs (ie, Supplemental Nutrition Assistance Program; Special Supplemental Nutrition Assistance Program for Women, Infants, and Children; emergency food box). One item asked participants to indicate whether they always, very often, often, sometimes, or never have enough food each month for their child to eat. At pre- and post-intervention, parents completed a survey composed of questions that assessed their attitudes (eg, confidence in their ability to cook vegetables) and behaviors (eg, trying new vegetables) related to the selected vegetables. Three items, adapted from previous surveys (25,26), were used to assess confidence in using various methods to cook vegetables: 1) How confident do you feel boiling or steaming vegetables? 2) How confident do you feel oven baking, roasting, or grilling vegetables? and 3) How confident do you feel pan-frying vegetables? Confidence was rated on a scale from 1 to 5 (not confident at all to very confident). Participants were also asked to rate on a scale from 1 to 5 (not confident at all to very confident) their level of confidence in preparing each of 10 vegetables. For this set of questions on confidence in preparation, 3 winter squashes (pumpkin squash, butternut squash, and delicata squash) were grouped together. We grouped these 3 squashes for the question on preparation because they are prepared similarly; we separated them for the questions on trying the vegetables and liking them because the vegetables have different flavors. To determine whether participants tried or liked the selected vegetables, we asked them to indicate whether they had ever tried each vegetable and if so, to rate their liking of each vegetable on a scale from 1 to 5 (strongly dislike to strongly like).

*Process evaluation.* Process evaluation included measures to assess home visitors’ fidelity to protocol and perception of intervention usability. To assess fidelity to protocol, home visitors submitted monthly logs of the intervention activities delivered to each family in their caseloads. Fidelity to protocol was measured as the proportion of parents who received the intervention components as planned. To assess home visitors’ perceptions of intervention usability, home visitors completed the Usage Rating Profile–Intervention (URP–I) (27) at post-intervention. The URP–I comprises 31 questions and 4 subscales: acceptability, understanding, feasibility, and systems support. Acceptability (12 questions) measures the degree to which an individual perceives that the intervention is an appropriate, fair, and reasonable way to address a problem. Understanding (8 questions) measures the degree to which an individual believes that he or she is knowledgeable about the intervention, including its purpose and how to implement it. Feasibility (5 questions) measures the extent to which an individual feels that the intervention could be implemented as prescribed given available time and resources. Systems support (6 questions) measures the degree to which an individual believes that the intervention requires systems support, including assistance from administrators or coworkers, to implement successfully. The 31 items were rated on a 5-point Likert-type scale from 1 to 5 (strongly disagree to strongly agree). 

### Statistical analyses

To maintain consistency in the dose of the home visit intervention, we restricted our analysis to participants without co-enrolled siblings because families with co-enrolled siblings receive longer home visits. Of the 104 participants meeting this criterion, only 58 provided both pre- and post-intervention data (a response rate of 56%).

We calculated the following descriptive statistics: means and standard deviations for continuous variables (ie, child’s and parent’s age) and numbers and percentages for categorical variables (ie, sex, ethnicity, race, employment status, educational attainment, participation in supplemental food assistance programs, and food security status).

Before analyzing the primary outcome variables, we first created binary categories for each outcome based on the 5-level Likert-response scales. We dichotomized these outcomes to reflect the overall goal of the program (to increase liking and confidence in preparing the selected vegetables) and to improve our ability to make statistical comparisons of the pre- and post-intervention results in our small samples. For the questions on confidence in preparing each vegetable, responses were dichotomized into “confident” (combining responses of 4 and 5 on the Likert scale) and “not confident” (combining the responses of 1, 2, and 3 on the Likert scale). The question asking whether participants liked each vegetable was dichotomized into “like” (combining the 2 categories of strongly like or like) and “dislike” (combining the 3 categories of neutral, dislike to strongly dislike). The proportions of respondents who were confident in preparing each vegetable, who had tried each vegetable, and who liked each vegetable were compared before and after the intervention. Because of small sample sizes of participants who had ever tried the vegetables pre-intervention, we were unable to make meaningful or statistical comparisons for the following 5 vegetables: turnip, rutabaga, parsnip, delicata squash, and spaghetti squash. We used McNemar χ^2^ tests to make comparisons (release 11, StataCorp LP). Significance was set at an α level of .05.

For the process evaluation, we summarized descriptive data as numbers and percentages of parents that participated in the intervention activities and mean and standard deviation (SD) to describe home visitors’ perceptions of intervention usability.

## Results

All 14 home visitors were women; one had 1 year or less of experience, 3 home visitors had 5 to 10 years of experience, and 10 home visitors had 11 or more years of experience. Two home visitors had an associate degree from a community college, 11 had a degree from a 4-year university, and one had a graduate degree.

At baseline, the mean age of enrolled children was 1.9 years and the mean age of parents was 29.5 years (Table 1). Nearly all parent participants were mothers. Most participants were white, unemployed, and participated in supplemental food assistance programs. Approximately 43% of respondents had less than 12 years of education. Most parents responded that they always or very often had enough food each month for their child to eat.

**Table 1:** Baseline Demographic Characteristics of Parents in a Early Head Start Home Visiting Program (N = 58) Who Participated in a Pilot Study to Test Feasibility of Using a Cooking Intervention to Improve Attitudes and Behaviors Related to Vegetable Consumption, Portland, Oregon, 2013–2014

Characteristic	No. of Respondents	Value^a^
**Child’s age, mean (SD), y**	57	1.9 (0.9)
**Parent’s age, mean (SD), y**	58	29.5 (7.5)
**Sex of parent**		
Female	58	55 (94.8)
Male		3 (5.2)
**Ethnicity**		
Non-Hispanic	47	12 (25.5)
Hispanic		35 (74.5)
**Race**		
White	35	26 (72.2)
Black		2 (8.3)
Other		7 (19.5)
**Employment status**		
Full-time	56	9 (16.1)
Part-time		2 (3.6)
Other^b^		45 (80.4)
**Educational attainment**		
<12 y	49	21 (42.9)
High school diploma/GED		13 (26.5)
Associate degree (2-year) or bachelor’s degree (4-year)		15 (30.6)
**Participation in supplemental food assistance programs**	58	58 (100.0)
**Parent has enough food each month for child to eat**		
Always	58	43 (74.1)
Very often		4 (6.9)
Often		7 (12.1)
Sometimes		4 (6.9)

Abbreviations: GED, general educational development; SD, standard deviation.

a All values are number (percentage) unless otherwise indicated. Percentages may not total 100 because of rounding.

b Seasonal, unemployed, in training or school, retired, or disabled.

The percentage of participants who were confident in baking, roasting, or grilling vegetables increased significantly from 60.7% (34 of 56) pre-intervention to 87.9% (51 of 58) post-intervention. We found no significant changes from pre-intervention to post-intervention for boiling/steaming (pre-intervention, 67.9% vs postintervention, 69.0%) or pan-frying vegetables (pre-intervention, 82.1% vs postintervention, 91.4%). We found significant increases in confidence pre-intervention to post-intervention in preparing 6 vegetables: turnips, rutabaga, parsnips, beets, sweet potatoes, and spaghetti squash (Table 2). The largest change was in the percentage of respondents who indicated confidence in preparing turnips; the percentage increased by more than 4 times, from 9.6% pre-intervention to 43.9% post intervention.

**Table 2:** Proportion of Respondents Who Felt Confident In Preparing Selected Vegetables Pre- and Post-Intervention, Pilot Program to Increase Vegetable Consumption in an Early Head Start Home Visiting Program, Portland, Oregon, 2013–2014

Intervention Group	No. of Respondents	No. (%) Who Felt Confident	*P* Value^a^
**Turnips**			
Pre	52	5 (9.6)	<.001
Post	57	25 (43.9)	
**Rutabaga**			
Pre	52	7 (13.5)	<.001
Post	56	22 (39.3)	
**Parsnips**			
Pre	51	6 (11.8)	<.001
Post	55	23 (41.8)	
**Carrots**			
Pre	56	53 (94.6)	.56
Post	58	56 (96.6)	
**Beets**			
Pre	53	18 (34.0)	<.001
Post	54	40 (74.1)	
**Sweet potatoes**			
Pre	55	37 (62.3)	.01
Post	58	49 (84.5)	
**Potatoes**			
Pre	57	53 (93.0)	>.99
Post	58	54 (93.1)	
**Cabbage**			
Pre	57	45 (79.0)	.06
Post	57	51 (89.5)	
**Pumpkin squash, butternut squash, or delicata squash**			
Pre	56	39 (69.6)	.13
Post	58	46 (79.3)	
**Spaghetti squash**			
Pre	53	19 (35.9)	<.001
Post	57	39 (68.4)	

a Determined by McNemar χ^2^ test.

We found significant increases pre-intervention to post-intervention in the percentage of people who tried the following 7 vegetables: turnips, rutabaga, parsnips, beets, butternut squash, spaghetti squash, and delicata squash (Table 3). The percentage of participants who tried rutabaga increased from 16.7% to 80.7%. 

**Table 3:** Proportion of Respondents Who Tried a Vegetable Pre- and Post-Intervention, Pilot Program to Increase Vegetable Consumption in an Early Head Start Home Visiting Program, Portland, Oregon, 2013–2014

Vegetable/Intervention Group	No. of Respondents	No. (%) Who Tried Item	*P* Value^a^
**Turnips**			
Pre	55	12 (21.8)	<.001
Post	58	51 (87.9)	
**Rutabaga**			
Pre	54	9 (16.7)	<.001
Post	57	46 (80.7)	
**Parsnips**			
Pre	55	12 (21.8)	<.001
Post	54	41 (75.9)	
**Carrots**			
Pre	57	56 (94.6)	.32
Post	58	56 (96.6)	
**Beets**			
Pre	56	33 (58.9)	<.001
Post	57	53 (93.0)	
**Sweet potatoes**			
Pre	55	52 (94.6)	.32
Post	58	57 (98.3)	
**Potatoes**			
Pre	58	58 (100.0)	NA
Post	56	56 (100.0)	
**Cabbage**			
Pre	58	57 (98.3)	>.99
Post	57	56 (98.3)	
**Pumpkin squash**			
Pre	57	52 (91.2)	.41
Post	56	53 (94.6)	
**Butternut squash**			
Pre	56	31 (55.4)	<.001
Post	57	52 (91.2)	
**Spaghetti squash**			
Pre	57	24 (42.1)	<.001
Post	58	47 (81.0)	
**Delicata squash**			
Pre	56	21 (37.5)	<.001
Post	58	44 (75.9)	

Abbreviation: NA, not applicable.

a Determined by McNemar χ^2^ test.

For the 7 vegetables for which we had adequate sample sizes at pre-intervention (carrots, beets, sweet potatoes, potatoes, cabbage, pumpkin squash, and butternut squash), the percentage of respondents who liked a vegetable increased significantly (*P* = .04) only for beets: 16 of the 33 (48.5%) participants who had tried beets at pre-intervention indicated they liked them, whereas 40 of the 53 (75.5%) participants who tried beets at post-intervention liked them. At pre-intervention, only 8 people had ever tried rutabaga, 10 people had ever tried turnips, 9 people had ever tried parsnips, 17 people had ever tried delicata squash, and 21 people had ever tried spaghetti squash. Among those participants who had never tried these vegetables, the proportion of participants who tried them during the intervention and liked them at post-intervention ranged from 37% (14/38) for turnips to 65% (17/26) for delicata squash.

On average, 96% of parents participated in the cooking activity each month and 94% participated in at least 1 complementary activity. Of the complementary activities, 81% participated in the literacy activity, 56% participated in the floor time activity, 26% in the art activity, and 11% in the music and movement activity. Twelve of 14 home visitors completed the usage survey post-intervention. Mean (SD) scores were 4.6 (0.3) for acceptability, 4.4 (0.4) for feasibility, 4.5 (0.4) for understanding, and 2.2 (0.3) for systems support.

## Discussion

To our knowledge, our study is the first study in which a cooking intervention was integrated into an existing home visiting program. The study showed significant increases in the proportion of parents who indicated confidence in baking, roasting, or grilling vegetables — the cooking methods emphasized in the intervention — and in the preparation of 6 of 10 selected vegetables. We observed no significant changes in confidence in preparing carrots, potatoes, cabbage, and 3 types of squash, but confidence levels for the preparation of these vegetables were already high pre-intervention (especially for carrots and potatoes). The finding that a cooking intervention delivered through a home visiting program had significant and positive effects on cooking confidence among participants is consistent with previous evaluations of cooking programs conducted in group settings with similar populations (11,12,14). Our study also showed that a cooking intervention in parents’ homes may encourage low-income parents of young children to taste foods they had not tasted before: post-intervention, we found significant increases in the proportion of parents who had tried 7 of the 12 selected vegetables.

Results from the process evaluation showed that the intervention was implemented successfully, with high fidelity to protocol. Almost all of the families participated in the monthly cooking activity. This high and consistent level of participation suggests that home visiting may be an effective mechanism for engaging low-income parents with young children in a cooking intervention. Of the 4 additional activities, the literacy activity was implemented with most (81%) families each month, whereas the floor time, art, music-and-movement activities were implemented with only 11% to 56% of families each month. Early Head Start Home Visiting requires home visitors to implement a monthly literacy activity (but not floor time, art or music-and-movement activities) with each family in their caseloads. Previous studies show that alignment between intervention activities and program performance standards is critical for successful intervention implementation (28).

Home visitors reported that the intervention was acceptable, feasible, and easy to understand. The low mean score for the systems-support subscale indicates that home visitors perceived that systems supports were needed to implement the intervention with high fidelity to protocol. Systems supports included training and on-going support from the home visitors’ immediate supervisor who purchased and distributed supplies, among other activities, to facilitate the successful implementation of the intervention.

Our pilot study has several limitations. First, this was a feasibility study conducted with parents and home visitors from a single Early Head Start Home Visiting program. The results, therefore, may not be generalizable to parents and home visitors participating in other programs. Second, our study lacked a control or comparison group. Third, because of resource constraints, we did not include vegetable consumption as an outcome measure. Fourth, social desirability response bias may have influenced survey findings.

Despite these limitations, this study has several strengths. First, in addition to helping to overcome barriers (eg, lack of transportation, unreliable work schedules) associated with group interventions (14,15) the weekly structure of the participating home visiting program provided multiple opportunities for home visitors to follow up with parents about intervention activities and to reinforce intervention messages. Second, implementing a cooking intervention in the home allowed parents to learn how to prepare vegetables using the kitchen tools and ingredients they had on hand. Third, integrating a cooking intervention through the existing channel of a home visiting program allowed us to minimize costs. Finally, we used process measures to learn lessons that can be applied to a follow-up study. In particular, we plan to reduce the number of intervention components to 2 activities — cooking and literacy. Focusing on these 2 activities will increase alignment between intervention activities and program performance standards, reduce administrator burden, and ensure that each family is exposed to the same intervention components. In future studies, we will also measure the amount of time home visitors spent implementing intervention activities, which may influence program outcomes.
